# Diethyl 2-(2-nitro­benzyl­idene)malonate

**DOI:** 10.1107/S1600536809032668

**Published:** 2009-08-22

**Authors:** S. Thenmozhi, S. Ranjith, A. SubbiahPandi, V. Dhayalan, A. K. MohanaKrishnan

**Affiliations:** a Department of Physics, Presidency College (Autonomous), Chennai 600 005, India; b Department of Organic Chemistry, University of Madras, Guindy Campus, Chennai 600 025, India

## Abstract

In the title compound, C_14_H_15_NO_6_, the ethoxy­carbonyl groups adopt extended conformations. In the crystal, mol­ecules are linked into  centrosymmetric dimers *via* pairs of C—H⋯O hydrogen bonds with a *R*_2_^2^(20) motif.

## Related literature

For biological activity of nitro­gen-containing building blocks derived from α-methyl­ene-β-hydr­oxy esters, see: Singh & Batra (2008 ▶); Masson *et al.* (2007 ▶); Basavaiah *et al.* (2003 ▶); Youngme *et al.* (2007 ▶); Ma *et al.* (2005 ▶); Soldatov *et al.* (2003 ▶); Hinckley (1969 ▶).
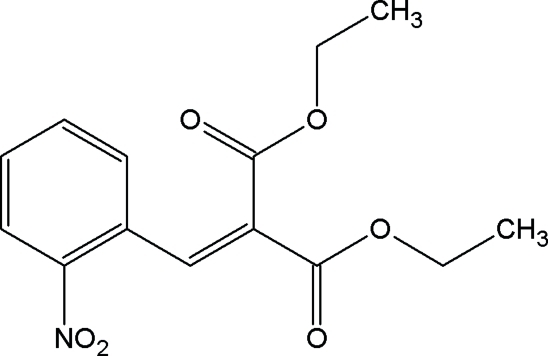


## Experimental

### 

#### Crystal data

C_14_H_15_NO_6_
*M* *_r_* = 293.27
Triclinic, 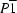

*a* = 7.8410 (2) Å
*b* = 8.5571 (2) Å
*c* = 12.3533 (4) Å
α = 80.866 (2)°
β = 75.037 (1)°
γ = 64.402 (1)°
*V* = 721.10 (3) Å^3^
*Z* = 2
Mo *K*α radiation
μ = 0.11 mm^−1^
*T* = 293 K
0.21 × 0.19 × 0.17 mm

#### Data collection

Bruker Kappa APEXII CCD diffractometer
Absorption correction: multi-scan (*SADABS*; Sheldrick, 1996 ▶) *T*_min_ = 0.978, *T*_max_ = 0.982
19570 measured reflections
4226 independent reflections
3259 reflections with *I* > 2σ(*I*)
*R*_int_ = 0.025

#### Refinement

*R*[*F* ^2^ > 2σ(*F* ^2^)] = 0.048
*wR*(*F* ^2^) = 0.145
*S* = 1.06
4226 reflections
192 parameters
H-atom parameters constrained
Δρ_max_ = 0.38 e Å^−3^
Δρ_min_ = −0.28 e Å^−3^

### 

Data collection: *APEX2* (Bruker, 2004 ▶); cell refinement: *SAINT* (Bruker, 2004 ▶); data reduction: *SAINT*; program(s) used to solve structure: *SHELXS97* (Sheldrick, 2008 ▶); program(s) used to refine structure: *SHELXL97* (Sheldrick, 2008 ▶); molecular graphics: *ORTEP-3* (Farrugia, 1997 ▶); software used to prepare material for publication: *SHELXL97* and *PLATON* (Spek, 2009 ▶).

## Supplementary Material

Crystal structure: contains datablocks global, I. DOI: 10.1107/S1600536809032668/bt5022sup1.cif

Structure factors: contains datablocks I. DOI: 10.1107/S1600536809032668/bt5022Isup2.hkl

Additional supplementary materials:  crystallographic information; 3D view; checkCIF report

## Figures and Tables

**Table 1:** Hydrogen-bond geometry (Å, °)

*D*—H⋯*A*	*D*—H	H⋯*A*	*D*⋯*A*	*D*—H⋯*A*
C2—H2⋯O3^i^	0.93	2.47	3.1683 (18)	132

Symmetry code: (i) 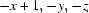.

**Fig. 1.**
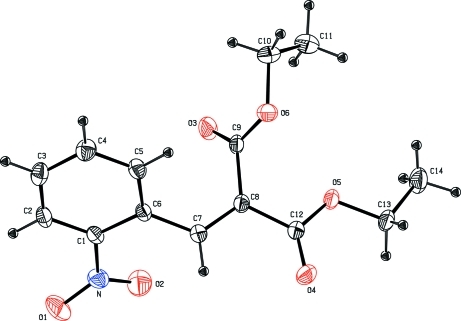
View of the title molecule with the atom labeling scheme. The displacement ellipsoids are drawn at the 30% probability level while the H atoms are shown as small spheres of arbitrary radii.

**Fig. 2.**
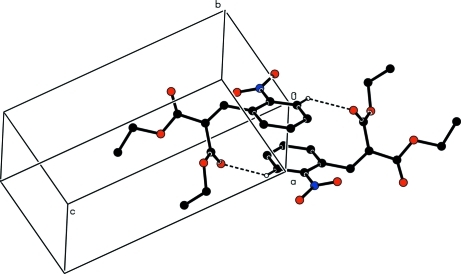
The crystal structure showing the centrosymmetric hydrogen bond motif R22(20). For the sake of clarity, the H atoms not involved in the motif have been omitted. The atoms marked with an asterisk (*) are at the symmetry position (1 - x, -y, -z). The dashed lines indicate the hydrogen bonds.

## supplementary crystallographic information

### Comment

Nitrogen-containing building blocks derived from a-methylene-β-hydroxy esters (Morita-Baylis-Hillman adducts) have been widely employed in modern organic chemistry for the synthesis of natural products and heterocycles of biological relevance (Singh & Batra, 2008; Masson *et al.*, 2007; Basavaiah *et al.*, 2003). β-Diketone as an excellent chelating group has been widely used in supramolecular chemistry. It can form a variety of complexes with various transition-metals (*e.g.* Cu, Co, Ni, Mn, Pd) or rare-earth metals (*e.g.* Eu, Sm, La, Gd) (Youngme *et al.*, 2007; Ma *et al.*, 2005). These metal complexes have significant applications in material science or act as chemical shift reagents (Soldatov *et al.*, 2003; Hinckley, 1969). In view of this importance, the crystal structure determination of the title compound (Fig.1) has been carried out.

A perspective view of the title compound   with the atom-numbering scheme is shown in Fig. 1. The deviations of the atoms N, O1 and O2 from the least-squares plane of the phenyl rings are -0.080 (1), 0.296 (2) and -0.527 (1) Å. The ethoxycarbonyl groups adopt extended conformation as can be seen from the torsion angles C8- C9- O6- C10 [175.6 (1)°], C9- O6- C10- C11 [-79.9 (2)°], C8- C12- O5- C13[178.6 (1)°] and C12- O5- C13- C14 [178.5 (1)°]. The C2–H2···O3 hydrogen bonds form a cyclic centrosymmetric dimer [*R*_2_^2^(20)] shown in Fig.2.

### Experimental

A mixture of 2-nitrobenzaldehyde (5 g, 33.11 mmol) in dry xylene (50 ml), ethylene diamine di acetate (1.2 g, 6.66 mmol) and diethyl malonate (6.36 g, 39.7 mmol) were added. The reaction was then refluxed for 12 h, it was then poured over ice - water (100 ml), extracted with CHCl_3_ (60 ml) and dried (Na_2_ SO_4_). The removal of solvent followed by recrystallization from methanol. Single crystals of the title compound suitable for X-ray diffraction were obtained by slow evaporation of a solution in methanol.

### Refinement

All H atoms were fixed geometrically and allowed to ride on their parent C atoms, with C–H distances fixed in the range 0.93–0.97 Å with *U*_iso_(H) = 1.5*U*_eq_(C) for methyl H 1.2*U*_eq_(C) for other H atoms.

### Crystal data

**Table:**

C_14_H_15_NO_6_	*Z* = 2
*M**_r_* = 293.27	*F*(000) = 308
Triclinic, *P*1	*D*_x_ = 1.351 Mg m^−^^3^
Hall symbol: -P 1	Mo *K*α radiation, λ = 0.71073 Å
*a* = 7.8410 (2) Å	Cell parameters from 4226 reflections
*b* = 8.5571 (2) Å	θ = 1.7–30.7°
*c* = 12.3533 (4) Å	µ = 0.11 mm^−^^1^
α = 80.866 (2)°	*T* = 293 K
β = 75.037 (1)°	Block, colourless
γ = 64.402 (1)°	0.21 × 0.19 × 0.17 mm
*V* = 721.10 (3) Å^3^	

### Data collection

**Table:**

Bruker Kappa APEXII CCD diffractometer	4226 independent reflections
Radiation source: fine-focus sealed tube	3259 reflections with *I* > 2σ(*I*)
graphite	*R*_int_ = 0.025
ω scans	θ_max_ = 30.2°, θ_min_ = 1.7°
Absorption correction: multi-scan (*SADABS*; Sheldrick, 1996)	*h* = −6→11
*T*_min_ = 0.978, *T*_max_ = 0.982	*k* = −11→12
19570 measured reflections	*l* = −17→17

### Refinement

**Table:**

Refinement on *F*^2^	Primary atom site location: structure-invariant direct methods
Least-squares matrix: full	Secondary atom site location: difference Fourier map
*R*[*F*^2^ > 2σ(*F*^2^)] = 0.048	Hydrogen site location: inferred from neighbouring sites
*wR*(*F*^2^) = 0.145	H-atom parameters constrained
*S* = 1.06	*w* = 1/[σ^2^(*F*_o_^2^) + (0.0732*P*)^2^ + 0.1321*P*] where *P* = (*F*_o_^2^ + 2*F*_c_^2^)/3
4226 reflections	(Δ/σ)_max_ < 0.001
192 parameters	Δρ_max_ = 0.38 e Å^−^^3^
0 restraints	Δρ_min_ = −0.28 e Å^−^^3^

### Special details

**Table:**

Geometry. All e.s.d.'s (except the e.s.d. in the dihedral angle between two l.s. planes) are estimated using the full covariance matrix. The cell e.s.d.'s are taken into account individually in the estimation of e.s.d.'s in distances, angles and torsion angles; correlations between e.s.d.'s in cell parameters are only used when they are defined by crystal symmetry. An approximate (isotropic) treatment of cell e.s.d.'s is used for estimating e.s.d.'s involving l.s. planes.
Refinement. Refinement of *F*^2^ against ALL reflections. The weighted *R*-factor *wR* and goodness of fit *S* are based on *F*^2^, conventional *R*-factors *R* are based on *F*, with *F* set to zero for negative *F*^2^. The threshold expression of *F*^2^ > σ(*F*^2^) is used only for calculating *R*-factors(gt) *etc*. and is not relevant to the choice of reflections for refinement. *R*-factors based on *F*^2^ are statistically about twice as large as those based on *F*, and *R*- factors based on ALL data will be even larger.

### Fractional atomic coordinates and isotropic or equivalent isotropic displacement parameters (Å^2^)

**Table:**

	*x*	*y*	*z*	*U*_iso_*/*U*_eq_	
C1	0.32734 (17)	0.26054 (16)	0.00483 (10)	0.0324 (3)	
C2	0.4208 (2)	0.18112 (18)	−0.09512 (11)	0.0393 (3)	
H2	0.3530	0.1563	−0.1367	0.047*	
C3	0.6156 (2)	0.1390 (2)	−0.13275 (12)	0.0445 (3)	
H3	0.6808	0.0854	−0.2002	0.053*	
C4	0.7138 (2)	0.1765 (2)	−0.07003 (12)	0.0444 (3)	
H4	0.8458	0.1469	−0.0951	0.053*	
C5	0.61807 (19)	0.25790 (18)	0.02979 (11)	0.0380 (3)	
H5	0.6867	0.2834	0.0704	0.046*	
C6	0.42104 (17)	0.30233 (16)	0.07069 (10)	0.0307 (2)	
C7	0.32275 (18)	0.40052 (17)	0.17243 (11)	0.0335 (3)	
H7	0.2096	0.4996	0.1694	0.040*	
C8	0.38277 (18)	0.35895 (16)	0.26840 (10)	0.0322 (3)	
C9	0.54181 (19)	0.18778 (16)	0.28991 (10)	0.0339 (3)	
C10	0.8427 (2)	0.0493 (2)	0.34618 (14)	0.0517 (4)	
H10A	0.9576	0.0709	0.3360	0.062*	
H10B	0.8725	−0.0413	0.2972	0.062*	
C11	0.7894 (3)	−0.0099 (2)	0.46438 (15)	0.0570 (4)	
H11A	0.7533	0.0818	0.5126	0.086*	
H11B	0.8980	−0.1092	0.4840	0.086*	
H11C	0.6824	−0.0408	0.4730	0.086*	
C12	0.27995 (18)	0.48151 (17)	0.36030 (11)	0.0350 (3)	
C13	0.2334 (2)	0.51216 (19)	0.55376 (11)	0.0432 (3)	
H13A	0.0935	0.5618	0.5646	0.052*	
H13B	0.2752	0.6061	0.5419	0.052*	
C14	0.2947 (3)	0.4002 (2)	0.65347 (13)	0.0581 (4)	
H14A	0.2440	0.3131	0.6678	0.087*	
H14B	0.2462	0.4697	0.7174	0.087*	
H14C	0.4334	0.3452	0.6397	0.087*	
N	0.12284 (17)	0.29396 (16)	0.04462 (12)	0.0446 (3)	
O1	0.03150 (19)	0.3042 (2)	−0.02475 (13)	0.0753 (4)	
O2	0.05294 (17)	0.30788 (19)	0.14427 (11)	0.0651 (4)	
O3	0.53708 (18)	0.05173 (13)	0.28408 (10)	0.0536 (3)	
O4	0.17045 (18)	0.63004 (14)	0.34780 (9)	0.0564 (3)	
O5	0.32173 (14)	0.40462 (12)	0.45770 (8)	0.0394 (2)	
O6	0.68593 (13)	0.20702 (12)	0.31542 (9)	0.0417 (2)	

### Atomic displacement parameters (Å^2^)

**Table:**

	*U*^11^	*U*^22^	*U*^33^	*U*^12^	*U*^13^	*U*^23^
C1	0.0360 (6)	0.0334 (6)	0.0324 (6)	−0.0158 (5)	−0.0140 (5)	0.0017 (5)
C2	0.0546 (8)	0.0390 (7)	0.0318 (6)	−0.0213 (6)	−0.0185 (6)	−0.0007 (5)
C3	0.0560 (8)	0.0465 (8)	0.0301 (7)	−0.0220 (7)	−0.0034 (6)	−0.0068 (6)
C4	0.0409 (7)	0.0516 (8)	0.0402 (7)	−0.0221 (6)	0.0000 (6)	−0.0065 (6)
C5	0.0384 (6)	0.0467 (7)	0.0357 (7)	−0.0220 (6)	−0.0098 (5)	−0.0037 (6)
C6	0.0364 (6)	0.0320 (6)	0.0264 (6)	−0.0150 (5)	−0.0105 (4)	0.0001 (4)
C7	0.0345 (6)	0.0339 (6)	0.0331 (6)	−0.0130 (5)	−0.0095 (5)	−0.0037 (5)
C8	0.0374 (6)	0.0303 (6)	0.0299 (6)	−0.0130 (5)	−0.0083 (5)	−0.0052 (5)
C9	0.0445 (6)	0.0313 (6)	0.0249 (6)	−0.0119 (5)	−0.0099 (5)	−0.0057 (4)
C10	0.0354 (7)	0.0476 (8)	0.0584 (10)	−0.0017 (6)	−0.0121 (6)	−0.0072 (7)
C11	0.0533 (9)	0.0496 (9)	0.0595 (10)	−0.0091 (7)	−0.0232 (7)	0.0035 (8)
C12	0.0382 (6)	0.0330 (6)	0.0333 (6)	−0.0120 (5)	−0.0089 (5)	−0.0059 (5)
C13	0.0523 (8)	0.0386 (7)	0.0336 (7)	−0.0118 (6)	−0.0064 (6)	−0.0129 (6)
C14	0.0844 (12)	0.0509 (9)	0.0335 (8)	−0.0210 (8)	−0.0129 (8)	−0.0067 (7)
N	0.0378 (6)	0.0457 (7)	0.0561 (8)	−0.0183 (5)	−0.0164 (5)	−0.0047 (6)
O1	0.0558 (7)	0.1031 (11)	0.0868 (10)	−0.0357 (7)	−0.0377 (7)	−0.0131 (8)
O2	0.0455 (6)	0.0896 (10)	0.0628 (8)	−0.0328 (6)	0.0022 (5)	−0.0185 (7)
O3	0.0800 (8)	0.0325 (5)	0.0580 (7)	−0.0194 (5)	−0.0361 (6)	−0.0042 (5)
O4	0.0665 (7)	0.0376 (6)	0.0441 (6)	0.0029 (5)	−0.0168 (5)	−0.0086 (5)
O5	0.0511 (5)	0.0328 (5)	0.0292 (5)	−0.0096 (4)	−0.0096 (4)	−0.0086 (4)
O6	0.0364 (5)	0.0370 (5)	0.0497 (6)	−0.0103 (4)	−0.0137 (4)	−0.0033 (4)

### Geometric parameters (Å, °)

**Table:**

C1—C2	1.3756 (19)	C10—O6	1.4566 (17)
C1—C6	1.3991 (16)	C10—C11	1.482 (2)
C1—N	1.4615 (17)	C10—H10A	0.9700
C2—C3	1.375 (2)	C10—H10B	0.9700
C2—H2	0.9300	C11—H11A	0.9600
C3—C4	1.377 (2)	C11—H11B	0.9600
C3—H3	0.9300	C11—H11C	0.9600
C4—C5	1.383 (2)	C12—O4	1.1998 (16)
C4—H4	0.9300	C12—O5	1.3244 (16)
C5—C6	1.3917 (17)	C13—O5	1.4512 (16)
C5—H5	0.9300	C13—C14	1.483 (2)
C6—C7	1.4680 (17)	C13—H13A	0.9700
C7—C8	1.3278 (17)	C13—H13B	0.9700
C7—H7	0.9300	C14—H14A	0.9600
C8—C12	1.4878 (17)	C14—H14B	0.9600
C8—C9	1.4967 (17)	C14—H14C	0.9600
C9—O3	1.1947 (16)	N—O2	1.2132 (18)
C9—O6	1.3283 (16)	N—O1	1.2222 (17)
			
C2—C1—C6	123.07 (12)	O6—C10—H10B	109.4
C2—C1—N	117.21 (11)	C11—C10—H10B	109.4
C6—C1—N	119.66 (12)	H10A—C10—H10B	108.0
C3—C2—C1	119.15 (12)	C10—C11—H11A	109.5
C3—C2—H2	120.4	C10—C11—H11B	109.5
C1—C2—H2	120.4	H11A—C11—H11B	109.5
C4—C3—C2	119.66 (13)	C10—C11—H11C	109.5
C4—C3—H3	120.2	H11A—C11—H11C	109.5
C2—C3—H3	120.2	H11B—C11—H11C	109.5
C3—C4—C5	120.72 (13)	O4—C12—O5	124.51 (12)
C3—C4—H4	119.6	O4—C12—C8	124.15 (12)
C5—C4—H4	119.6	O5—C12—C8	111.33 (10)
C4—C5—C6	121.29 (12)	O5—C13—C14	107.61 (12)
C4—C5—H5	119.4	O5—C13—H13A	110.2
C6—C5—H5	119.4	C14—C13—H13A	110.2
C5—C6—C1	116.11 (11)	O5—C13—H13B	110.2
C5—C6—C7	119.28 (11)	C14—C13—H13B	110.2
C1—C6—C7	124.41 (11)	H13A—C13—H13B	108.5
C8—C7—C6	125.13 (11)	C13—C14—H14A	109.5
C8—C7—H7	117.4	C13—C14—H14B	109.5
C6—C7—H7	117.4	H14A—C14—H14B	109.5
C7—C8—C12	118.91 (11)	C13—C14—H14C	109.5
C7—C8—C9	122.12 (11)	H14A—C14—H14C	109.5
C12—C8—C9	118.84 (10)	H14B—C14—H14C	109.5
O3—C9—O6	125.05 (12)	O2—N—O1	123.34 (13)
O3—C9—C8	123.16 (12)	O2—N—C1	118.78 (11)
O6—C9—C8	111.79 (11)	O1—N—C1	117.87 (13)
O6—C10—C11	111.16 (12)	C12—O5—C13	116.56 (10)
O6—C10—H10A	109.4	C9—O6—C10	116.65 (11)
C11—C10—H10A	109.4		
			
C6—C1—C2—C3	0.5 (2)	C7—C8—C9—O6	123.02 (13)
N—C1—C2—C3	−176.61 (12)	C12—C8—C9—O6	−61.15 (15)
C1—C2—C3—C4	0.1 (2)	C7—C8—C12—O4	−15.0 (2)
C2—C3—C4—C5	−0.7 (2)	C9—C8—C12—O4	169.01 (14)
C3—C4—C5—C6	0.8 (2)	C7—C8—C12—O5	163.66 (12)
C4—C5—C6—C1	−0.28 (19)	C9—C8—C12—O5	−12.30 (16)
C4—C5—C6—C7	−175.29 (12)	C2—C1—N—O2	155.65 (14)
C2—C1—C6—C5	−0.36 (19)	C6—C1—N—O2	−21.53 (19)
N—C1—C6—C5	176.64 (11)	C2—C1—N—O1	−23.42 (19)
C2—C1—C6—C7	174.37 (12)	C6—C1—N—O1	159.40 (14)
N—C1—C6—C7	−8.63 (18)	O4—C12—O5—C13	−2.7 (2)
C5—C6—C7—C8	−50.59 (19)	C8—C12—O5—C13	178.64 (11)
C1—C6—C7—C8	134.84 (14)	C14—C13—O5—C12	178.49 (13)
C6—C7—C8—C12	173.32 (11)	O3—C9—O6—C10	−4.6 (2)
C6—C7—C8—C9	−10.8 (2)	C8—C9—O6—C10	175.61 (11)
C7—C8—C9—O3	−56.78 (19)	C11—C10—O6—C9	−79.96 (17)
C12—C8—C9—O3	119.05 (15)		

### Hydrogen-bond geometry (Å, °)

**Table:**

*D*—H···*A*	*D*—H	H···*A*	*D*···*A*	*D*—H···*A*
C2—H2···O3^i^	0.93	2.47	3.1683 (18)	132

Symmetry codes: (i) −*x*+1, −*y*, −*z*.
